# Acute effects of foam rolling vs. passive rest following a single bout of complex contrast training

**DOI:** 10.1007/s00421-026-06188-8

**Published:** 2026-03-24

**Authors:** Coşkun Rodoplu, Josef Fischer, Christian Burger, Mert Arabacı, Hüseyin Topçu, Ufuk Şekir, Ramiz Arabacı, Andreas Konrad

**Affiliations:** 1 Department of Common Courses, Bursa Technical University, Bursa, Turkey; 2 Institute of Human Movement Science, Sport and Health, Graz University, Mozartgasse 14, 8010 Graz, Austria; 3 Department of Physical Education and Sports Teaching, Bursa Uludağ University, Bursa, Turkey; 4 Department of Sport Medicine, Bursa Uludağ University, Bursa, Turkey

**Keywords:** Acute recovery, Muscle activity, Muscle performance, Y-balance test

## Abstract

**Purpose:**

A single session of complex contrast training (CCT) can induce lower-limb fatigue, making the optimization of post-CCT recovery strategies essential. Foam rolling (FR) is widely used and is believed to enhance acute recovery. Therefore, this study examined the acute effects of FR compared with passive recovery (PR) following a single bout of CCT on dynamic balance (YBT), static balance (SLST), horizontal jump performance (SLHD), vertical jump performance (CMJ), and muscle excitation measured via surface electromyography (sEMG).

**Methods:**

Twenty-one active males completed the study using a crossover design, being randomly allocated to FR and PR conditions. Assessments were performed at three time points: pre-test, immediately post-CCT (mid-test), and after the recovery intervention (post-test). Participants performed the CCT protocol prior to recovery (5 sets × 6 repetitions at 80% and 30% of 1RM). Test measures included YBT, SLST, SLHD, CMJ, and sEMG recordings from selected lower-limb muscles.

**Results:**

FR resulted in significant acute improvements in YBT, SLST, and SLHD compared with PR (p < 0.05), whereas no meaningful differences were detected in CMJ performance (p > 0.05). Although time effects were observed in sEMG excitation for some muscles (p < 0.05), no intervention × time interactions were detected (p > 0.05).

**Conclusions:**

FR is an effective acute recovery strategy for enhancing dynamic and static balance as well as horizontal jump performance following CCT. However, its influence on vertical jump performance and muscle excitation appears limited. Future research should incorporate broader neuromuscular assessments, varied FR protocols, and diverse populations to better clarify underlying mechanisms and optimize recovery outcomes.

**Supplementary Information:**

The online version contains supplementary material available at 10.1007/s00421-026-06188-8.

## Introduction

Resistance training plays a fundamental role in enhancing physical performance, reducing the risk of injury, and improving functional capacities such as balance, strength, and power (Fragala et al. 2019). Complex contrast training (CCT), which aims to improve functional capacity, involves the consecutive execution of two exercises that share a similar movement pattern but differ in load and velocity characteristics (e.g., high- and low-loaded half squats). Unlike traditional resistance training or isolated power exercises, CCT combines heavy and rapid loading within the same session, eliciting simultaneous mechanical and neuromuscular adaptations. This approach stimulates a broad spectrum of motor units within the lower-limb musculature (Barra-Moura et al. 2024) and induces a complex fatigue profile encompassing both mechanical (eccentric actions and the stretch–shortening cycle) and metabolic components (Cormier et al. 2020; Thapa et al. 2024). A single CCT session may elicit transient neuromuscular changes, which can be quantified using surface electromyography (sEMG). This approach allows for the assessment of whether specific exercises elicit differential levels of neural activation and whether these activation patterns evolve over time (Fischer et al. 2025a; 2025b; Petrigna et al. 2025).

In both sports and rehabilitation contexts, the combination of sEMG data with balance and jump tests is commonly used to evaluate individuals’ performance levels and injury risks. Balance is usually measured with tests where a person stands or reaches on one leg, while jumping ability is measured using single-leg hop for distance (SLHD) or vertical jump tests, often together with sEMG (Lazaridis et al. 2013; Bhanot et al. 2019; Kotsifaki et al. 2022; García-Arrabé et al. 2024). Integrating sEMG with these tests provides insight into muscle excitation patterns and neuromuscular control, allowing to detect compensations or asymmetries that may influence performance or injury susceptibility. Previous studies on long-term CCT have reported that it can enhance balance and jump performance (Kumar et al. 2023; Barra-Moura et al. 2024). However, evidence regarding the effects of a single bout of CCT remains limited (Maio Alves et al. 2010), and the fatigue or neuromuscular stress it induces may affect acute performance (Cormier et al. 2020). Therefore, implementing appropriate recovery strategies following CCT is essential for maintaining performance and preserving neuromuscular balance (Bhanot et al. 2019; Kotsifaki et al. 2022; Thapa et al. 2024).

Passive recovery (PR), stretching exercises, hydrotherapy (cold or hot water immersion), and massage are commonly used methods for promoting acute post-exercise recovery (Pablos et al. 2020; Konrad et al. 2022a). In recent years, foam rolling (FR) has gained attention as a practical, low-cost, and field-appropriate recovery technique. The proposed mechanisms underlying FR include changes in soft-tissue viscoelasticity, increased local blood circulation, modulation of nociceptive input, and reorganization of mechano- and proprioceptive afferent signalling (Cavanaugh et al. 2017; Wiewelhove et al. 2019). Through these mechanisms, FR may influence movement quality at both mechanical and neurophysiological levels compared with passive rest (Kasahara et al. 2022; Nakamura et al. 2021, 2022). The literature generally indicates that a single FR session increases joint flexibility without impairing muscle performance (Reiner et al. 2021; Szajkowski et al. 2025; Rodoplu et al. 2025). However, its effects on balance and jump performance appear to vary depending on study design and the specific tests used. Some studies have reported short-term improvements in y-balance test (YBT) and single-leg stance test (SLST) scores (Lee et al. 2018; de Benito et al. 2019; Casado et al. 2025; Chen et al. 2022; Wang et al. 2022), whereas results for vertical jump performance have been mixed, showing positive, negligible, or even negative effects (Healey et al. 2014; D’Amico et al. 2020; Zhang et al. 2020; Konrad et al. 2022b; Sedano and Maroto-Izquierdo 2025). Research investigating horizontal single-leg jump performance (e.g., SLHD) remains scarce (Nygaard Falch et al. 2020). Further, to better understand the effects of FR on performance, it is necessary to assess physical performance outcomes simultaneously to sEMG responses (Junker and Stöggl 2019; Nygaard Falch et al. 2020).

Regarding the neuromuscular responses of the lower-limb muscles following FR, the existing findings are inconsistent. Some studies have reported increased sEMG excitation and enhanced neuromuscular efficiency following FR (Bradbury-Squires et al. 2015; Lim et al. 2019), whereas others have observed reductions or no significant effects (Cavanaugh et al. 2017; Macgregor et al. 2018; MacDonald et al. 2014; Reiner et al. 2021). This inconsistency may partly stem from the inherent difficulty of interpreting sEMG signals during complex tasks such as balance assessments. Moreover, sEMG data collected during balance tasks pose additional interpretative challenges, as they require the dissociation of motor control, postural tone, and reflex contributions. Notably, pre-excitation and eccentric contraction phases have been identified as critical periods for analysis (da Silva et al. 2022; Mandalidis and Karagiannakis 2020). Consequently, there is a need for studies that concurrently examine the neuromuscular effects of FR during balance and jumping tasks using synchronized performance testing and sEMG recordings.

CCT has primarily been investigated as a long-term training method, with chronic applications reported to enhance both strength and balance performance (Cormier et al. 2020; Thapa et al. 2024), while evidence on the acute effects of CCT is lacking. As a recovery strategy, FR has gained substantial attention in the field of performance and recovery manipulation (Kasahara et al. 2022; Nakamura et al. 2021, 2022). However, to the best of our knowledge, no study has directly examined the acute effects of FR performed following a single bout of CCT on performance tests such as the YBT, SLST, SLHD, and countermovement jump (CMJ), along with corresponding sEMG outcomes. Thus, we aimed to investigate the acute effects of FR compared to PR following a single bout of CCT on YBT, SLST, SLHD, and CMJ performances including sEMG activities. We hypothesized that, compared with PR, FR would preserve or slightly enhance acute physical performance, while facilitate a faster normalization of lower-limb muscle excitation, particularly in the quadriceps, hamstrings, and calf muscles assessed with sEMG.

## Materials and methods

### Participants

A priori sample size calculation was performed using the G*Power 3.1.9.7 Software (Düsseldorf, Germany), based on a similar study conducted by Heinke et al. (2025). The parameters were set as follows: f = 0.40, α = 0.05 (5% probability of Type I error), and β = 0.80 (80% statistical power). The analysis indicated that a minimum of 18 participants would be sufficient for a crossover study design. Hence, a total of 21 healthy male participants aged between 20 and 25 years were recruited for the study. The inclusion criteria were as follows: (a) engaging in regular moderate-intensity physical activity at least three days per week, and (b) being eligible for exercise participation according to the Exercise Readiness Questionnaire for Everyone (EGZ-A +). The exclusion criteria included: (a) having any neurological or muscular disorder, (b) using stimulant substances, (c) taking medications that could affect physical performance prior to testing, and (d) having experienced any musculoskeletal injury affecting performance within the last three months.

### Study design

**Fig. 1:**
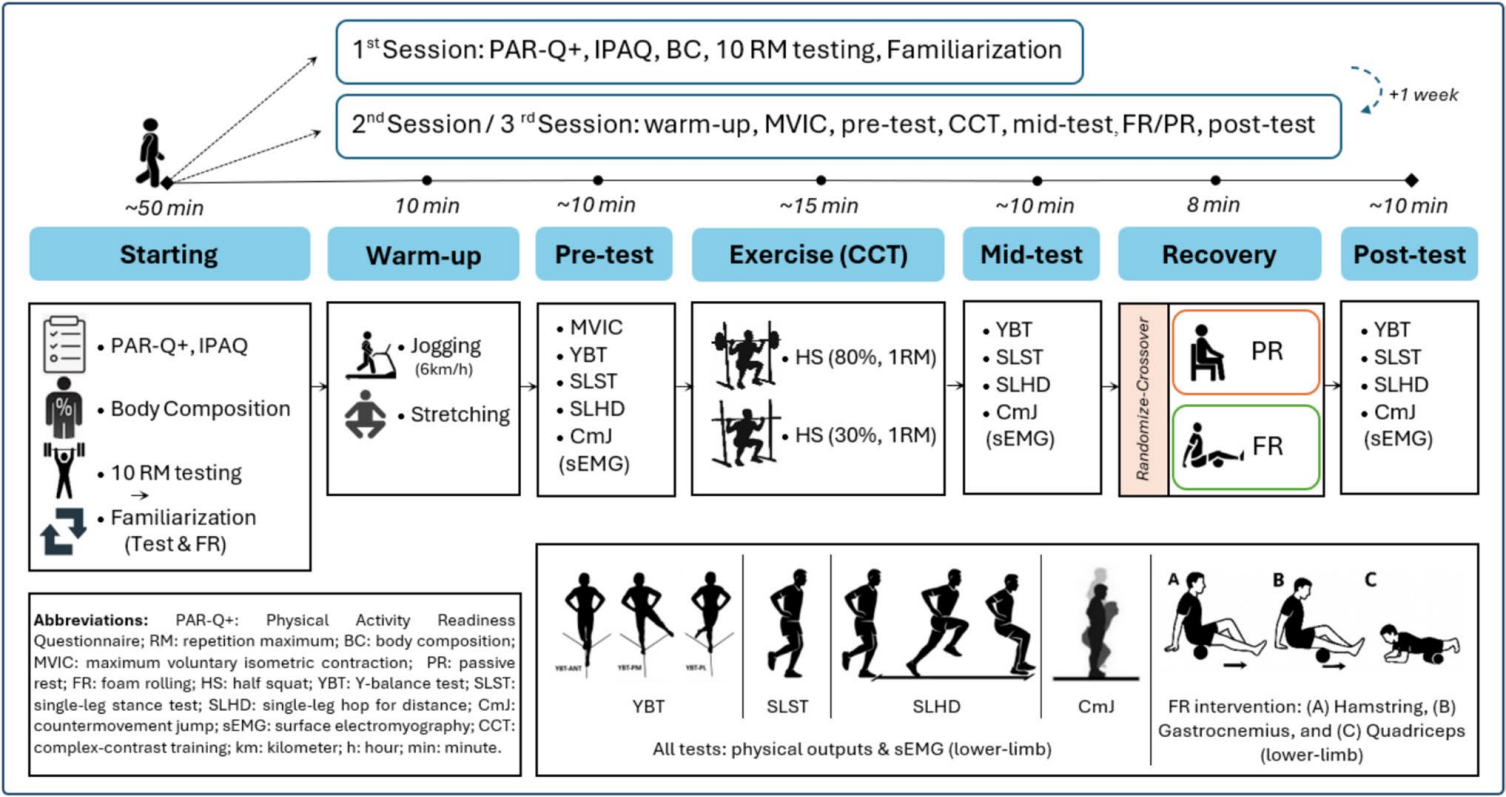
Design of the study

All participants visited the laboratory on three separate occasions, with at least one week between sessions, and were randomly assigned (via an online software program, www.randomizer.org) to either the FR or PR condition. During the first session (familiarization), participants’ height, weight, and body composition were assessed using a bioelectrical impedance analyzer (Tanita BC-418MA, Tokyo, Japan). A 10-repetition maximum (10RM) test was performed to determine exercise intensity for CCT, from which individual 1RM values were calculated. Participants were familiarized with the SLST, YBT, SLHD, and CMJ tests, as well as with the CCT protocol and FR intervention to ensure procedural consistency. In the second and third sessions, participants completed a standardized warm-up (5 min running at 6 km/h on a treadmill followed by 5 min dynamic stretching), MVIC (maximum voluntary isometric contraction) for normalization of the EMG, pre-test, CCT protocol (high-load: 80% 1RM; low-load: 30% 1RM), mid-test, recovery intervention (FR or PR), and post-test. Post-test measurements were initiated immediately after completion of the recovery intervention. The CCT protocol was monitored using the Borg Rating of Perceived Exertion (RPE) scale (6–20). The term “test” refers to the SLST, YBT, SLHD, and CMJ assessments, during which both physical performance and muscle excitation were recorded using a surface sEMG system (Ultium EMG, Noraxon USA Inc., Scottsdale, AZ, USA). Participants were instructed to refrain from strenuous exercise for 48 h before each session. All sessions were performed at the same time of day under controlled laboratory conditions (20–22 °C). The study was conducted in accordance with the Declaration of Helsinki and approved by the Ethics Committee of Bursa Uludağ University (Date: 16/01/2023; No: 2023–1/41; ID: 2011-KAEK-26/45) (Fig. 1).

### Procedures

#### Tests

##### Single-leg stance test (SLST)

This test was used to assess the participants’ static balance. It was performed as a single-leg balance test measuring the time a participant could maintain balance on the dominant (kicking) leg. Participants stood barefoot on one leg with their hands on their hips, and the duration from the moment they achieved balance until the first loss of postural stability (e.g., noticeable sway, foot movement, or touching the raised leg to the ground) was recorded. The test was repeated three times on the dominant leg with eyes closed, and the mean value was used for analysis (Liao et al. 2001).

##### Y-balance test (YBT)

This test evaluated the participants’ lower-limb dynamic balance and functional performance. While standing barefoot on the dominant leg with hands on the hips, participants were instructed to reach as far as possible with the contralateral leg in the anterior (ANT), posteromedial (PM), and posterolateral (PL) directions. During each trial, the supporting foot was kept stationary while maintaining balance and controlled movement. Distances were measured using a tape measure fixed to the floor. Each direction was tested three times, and the mean reach distance (in cm) was recorded. The YBT composite reach distance was calculated as the sum of the average reach distances in the three directions divided by leg length and multiplied by 100 (Shaffer et al. 2013). All data were normalized to leg length for statistical analysis (Wang et al. 2022).

##### Single-leg hop for distance (SLHD)

This test assessed horizontal jump performance on one leg. Participants stood barefoot with their hands on their hips and were instructed to perform a maximal forward hop from a marked starting line using the dominant leg, aiming to achieve the farthest possible distance while maintaining balance upon landing. Distances were measured using a measuring tape fixed to the floor. Each leg was tested three times, and the mean value (in cm) was used. All data were normalized to leg length (Sawle et al. 2017).

##### Countermovement jump (CMJ)

Vertical jump performance was measured using a Sport Expert™ MPS-501 device (Tümer Elektronik LTD., Turkey) with a precision of 0.1 cm. Jump height was calculated based on flight time. Participants stood barefoot with their hands on their hips and performed a maximal vertical jump, initiating the movement from an upright position to a semi-squat position (approximately 90° knee flexion) without any preparatory arm swing. Each participant performed three trials with 15 s of rest between them, and the mean jump height (in cm) was used for statistical analysis*.*

#### Surface electromyography (sEMG), root mean square (RMS) processing, and normalization

sEMG activity was recorded from the dominant leg using a portable 16-channel surface sEMG system (Ultium EMG, Noraxon USA Inc., Scottsdale, AZ). Differential bipolar surface electrodes (Ag/AgCl) were placed parallel to the muscle fibers on the quadriceps muscles (RF: Rectus Femoris, VM: Vastus Medialis, VL: Vastus Lateralis), the hamstring muscles (BF: Biceps Femoris, ST: Semitendinosus), and the medial gastrocnemius (GM). Electrode placement followed the SENIAM guidelines and anatomical references for innervation zones with an interelectrode distance of approximately 20 mm (Hermens et al. 2000). To minimize skin impedance, the electrode sites were shaved, cleaned with alcohol, and verified through a contraction test prior to placement.

MVIC measurements for normalization were obtained as follows: for the quadriceps, participants performed maximal knee extension against a dynamometer in a seated position with the knee at 60° flexion; for the hamstrings, maximal knee flexion was performed against manual resistance in a prone position with the knee at 30° flexion; and for the gastrocnemius, participants performed a heel raise while standing and lightly touching a table with two fingers for balance. Verbal encouragement was provided to ensure maximal effort, and three trials were conducted before each session. The mean of the highest 3-s RMS values was used as the reference MVIC (Burden 2010). For all tests, the mean RMS values obtained from three trials for each muscle were normalized to these MVIC values and expressed as %MVIC.

During all tests, sEMG signals of the quadriceps, hamstrings, and gastrocnemius were recorded using the integrated sEMG system. RMS values were calculated for each muscle during the specific phases of the tests: the balance-maintenance period in the SLST, the 2-s reaching (eccentric) phase in the YBT, and the push-off phase in the SLHD and CMJ. The reaching phase in the YBT was defined as the period from the onset of single-leg stance to maximum reach, while the push-off phase in the SLHD and CMJ was defined as the period from maximum knee flexion to take-off. These phases were identified using the integrated video/camera recording of the sEMG system (Bhanot et al. 2019).

All sEMG signals were recorded at a sampling rate of 2000 Hz using the myoResearch 4.0 software (Noraxon USA Inc., Scottsdale, AZ, USA). A band-pass filter of 10–500 Hz was applied to the recordings, and the RMS values of the signals were smoothed using a 150 ms moving average window. Video recordings were analyzed in slow motion to identify the push-off and take-off phases during the YBT, SLHD, and CMJ tests, and the corresponding sEMG signals were extracted. The processed data were exported to Microsoft Excel (Microsoft Corp., Redmond, WA, USA) and normalized to each muscle’s MVIC. All normalized sEMG data were subsequently transferred to IBM SPSS Statistics 27.0 software (IBM Corp., Armonk, NY, USA) for further statistical analysis.

#### Estimation of one-repetition maximum (1RM)

During the familiarization session, following a 10-min general warm-up, participants performed a 10RM test using a guided Smith machine at a standardized half-squat exercise corresponding to a 90° knee angle. Depth of the half-squat exercise was controlled with a manual goniometer and an adjustable tripod bar, and participants were instructed to descend until their hips lightly touched a bar. Based on observations during the warm-up and participant feedback, an approximate load corresponding to the 10RM was selected, and participants performed repetitions until momentary muscular failure. The repetition tempo was standardized using a metronome at four seconds per repetition (two seconds eccentric, two seconds concentric). The 1RM values were then estimated using the Brzycki equation (Brzycki 1993).

#### The CCT protocol

The CCT protocol consisted of performing the half-squat exercise sequentially with high (80% of 1RM) and low (30% of 1RM) loads. The high-load exercises were performed before the low-load, high-velocity exercises to ensure predominant activation of force-producing motor units followed by velocity-specific neural stimulation across the force–velocity spectrum (Cormier et al. 2020; Thapa et al. 2024). High-load squats were executed under metronome control at a slow, controlled tempo (2 s eccentric, 2 s concentric), while low-load squats were performed explosively at maximal velocity. Each exercise was completed for 5 sets of 6–8 repetitions, with the total duration of the protocol lasting approximately 15 min. To minimize fatigue-related declines in performance and reduce potential injury risk (Faigenbaum et al. 2006), a 2-min rest interval was provided between sets to ensure adequate recovery (de Salles et al. 2010). The detailed exercise protocol is presented in Table 1.

**Table 1:** Details of the CCT protocol

Set	HS-High Load (80% 1RM)	Rest Interval (~ sec)	HS-Low Load (30% 1RM)	Rest (min)	Duration (~ min)
1	HS × 6 reps	10–15	HS × 6–8 reps	2	3
2	HS × 6 reps	10–15	HS × 6–8 reps	2	3
3	HS × 6 reps	10–15	HS × 6–8 reps	2	3
4	HS × 6 reps	10–15	HS × 6–8 reps	2	3
5	HS × 6 reps	10–15	HS × 6–8 reps	2	3
Total	30 reps	~ 50–75 s	30–40 reps	10 min	~ 15 min

*HS* half-squat; *reps* repetitions; *1RM* 1 repetition maximal effort

#### Recovery

**Fig. 2:**
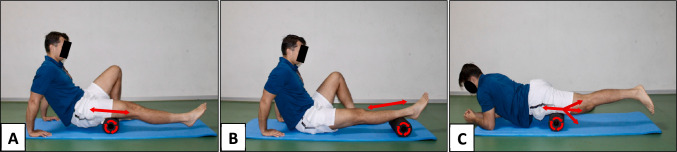
FR intervention protocols. (The red arrows in the figure illustrate the rhythmic back-and-forth motion of the foam roller, moving from the lower (distal) to the upper (proximal) part of the thigh and back again. This description refers to images (**A**) and (**B**), while image (**C**) shows three rolling regions that include the rectus femoris, vastus medialis and vastus lateralis muscles. The red circles indicate the rolling movement along the muscle, highlighting the specific areas targeted during foam rolling)

The recovery protocol was applied in two different conditions: PR and FR. In the PR condition, participants remained seated and motionless on a chair for 8 min. In the FR condition, participants used a foam roller (Blackroll, Bottighofen, Switzerland) to perform rhythmic rolling on the hamstrings, quadriceps, and gastrocnemius muscles, beginning with the dominant leg and then repeating the same sequence on the non-dominant leg. Each muscle group was rolled for 30 s, performed in two sets (total 60 s), with one complete rolling cycle (from distal to proximal and back) every 2 s (Behm et al. 2020). During the hamstrings and quadriceps rolling, participants were instructed to maintain a perceived pressure intensity of 7 out of 10 on the visual analog scale (VAS-10) (Behm et al. 2020). When the perceived pressure fell below this level, the researcher applied additional downward pressure to the leg. For the gastrocnemius muscles, rolling was performed using body weight only, without additional external pressure. The total rolling time for all muscle groups on both legs was approximately 6 min, when including transitions and electrode placements for the post-test, the overall duration of the intervention was about 8 min. The FR intervention is illustrated in Fig. 2.

### Statistical analyses

Descriptive statistics are presented as mean and standard deviation. The Shapiro–Wilk test confirmed that all data were normally distributed. A two-way repeated measures ANOVA was conducted to compare time and group interactions [two interventions (FR, PR) × three time points (pre-test, mid-test, post-test)], and pairwise comparisons were made. This ANOVA procedure was applied separately to the physical performance outcomes of each test (YBT, SLST, SLHD, CMJ) and to the sEMG activity of each muscle (RF, VL, VM, BF, ST, GM) within each test to examine time and group effects. Effect sizes were calculated as partial eta square (${\upeta }_{\mathrm{p}}^{2})$ for ANOVA (Cohen 1988), with values above 0.01, 0.06, and 0.14 indicating “small”, “medium”, and “large” effect sizes, respectively. Bonferroni correction was applied for all the post-hoc tests. The magnitude of significant pairwise differences was evaluated using Cohen’s d^z^ effect size (d^z^ = t/√n) and interpreted using standard thresholds: trivial < 0.20, small 0.20–0.59, moderate 0.60–1.19, large 1.20–1.99, and very large ≥ 2.0 (Hopkins et al. 2009). The significance level was set as p < 0.05.

## Results

**Fig. 3:**
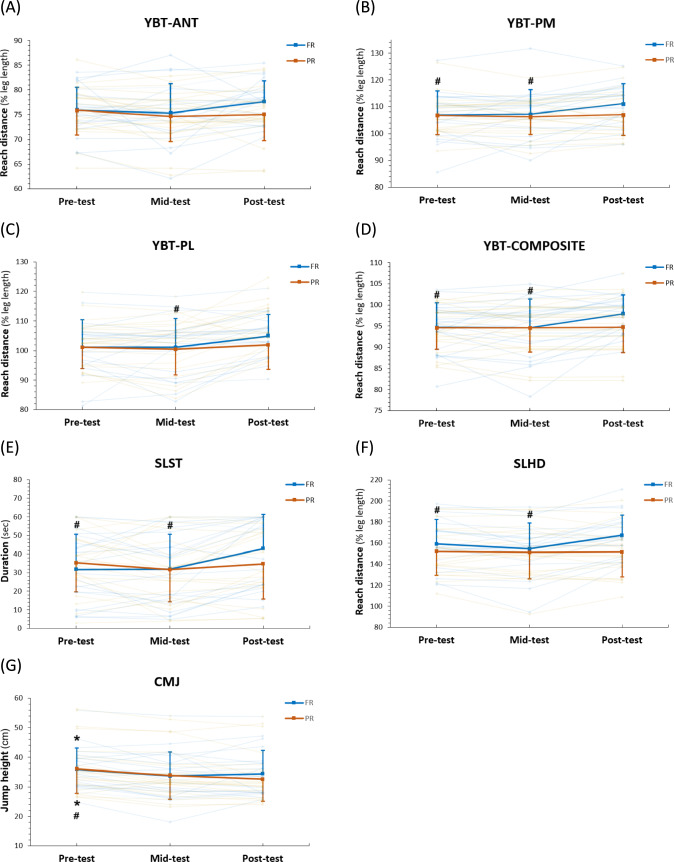
Mean ± SD physical performance outcomes for the FR and PR groups at pre-test, mid-test, and post-test time points. Panels show (**A**) YBT-ANT, (**B**) YBT-PM, (**C**) YBT-PL, (**D**) YBT composite score, (**E**) SLST duration, (**F**) SLHD normalized to leg length, and (**G**) CMJ height (cm).

**Fig. 4:**
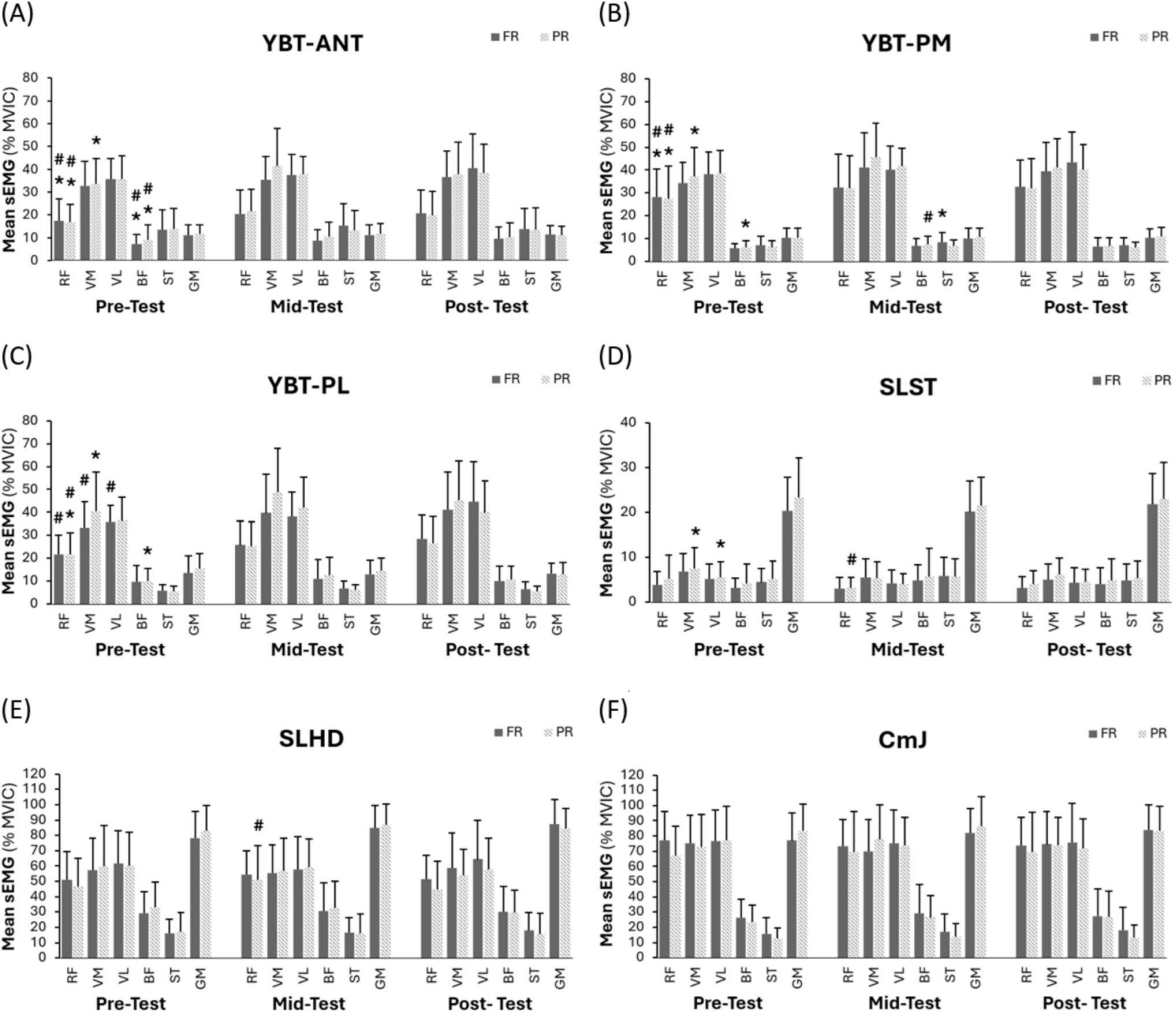
Mean ± SD EMG activity of the RF, VM, VL, BF, ST, and GM muscles for the FR and PR groups at pre-test, mid-test, and post-test time points. Panels show EMG activity (**A**) during the reaching phase in YBT-ANT, (**B**) in YBT-PM, (**C**) in YBT-PL, (**D**) throughout the stance phase in SLST, (**E**) during the propulsion phase in SLHD, and (**F**) during the propulsion phase in CMJ. Significant within-group pairwise comparisons from post-hoc tests are indicated by symbols (*p < 0.05, significantly different from mid-test; ^#^significantly different from post-test)

The anthropometrics of the participants are presented in Table 2. The mean ± SD values of all tests performed at the three different time points (pre-test, mid-test, and post-test), as well as the results of the ANOVA and significant pairwise comparisons, are presented in Table 3 and Fig. 3 while the sEMG activity of the RF, VM, VL, BF, ST, and GM muscles during these tests is shown in Fig. 4 and S1 File. The Borg scores immediately after a single bout of CCT were 18.1 ± 0.9 for the FR group and 18.3 ± 0.5 for the PR group.

**Table 2:** Descriptive statistics of the participants

Variable	Mean (SD)	Min	Max
Age (years)	21.5 (1.4)	20.0	25.0
Height (cm)	181.6 (7.2)	173.0	200.0
Weight (kg)	75.0 (11.0)	57.4	101.2
BMI (kg/m^2^)	22.7 (2.5)	18.1	27.2
FAT (%)	8.3 (5.1)	2.8	18.7
FM (kg)	6.5 (4.9)	1.7	16.7
FFM (kg)	68.4 (7.6)	55.5	84.5
TBW (kg)	50.0 (5.6)	40.6	61.9
1 RM (%)	95.6 (23.2)	60.0	169.6
PAL (MET-min/wk.)	1062.0 (573.5)	330.0	2088.3

*BMI*, body mass index; *FAT*, total body fat; *FM*, fat mass; *FFM*, fat-free mass; *TBW*, total body water; *RM*, repetition maximum; *PAL*, physical activity level

**Table 3:** Physical performance outcomes of participants: means, confidence intervals, and ANOVA analysis

Variable	Time	FR		PR		Intervention X Time			Time		
		Mean (SD)	[95% CI]	Mean (SD)	[95% CI]	F _(2,40)_	*p*	${\eta }_{p}^{2}$	F _(2, 40)_	*p*	${\eta }_{p}^{2}$
YBT (%)	Pre-test	75.89 (4.58)	73.81–77.98	75.87 (4.96)	73.61–78.13	3.095	0.056	0.134^ȶȶ^	2.805	0.072	0.123^ȶȶ^
ANT	Mid-test	75.33 (5.90)	72.64–78.02	74.69 (5.14)	74.69–72.34						
	Post-test	77.60 (4.18)	75.70–79.51	75.07 (5.30)	72.66–77.49						
	Pre-test	106.95 (8.88)§	96.83–105.37	106.87 (7.13)	103.62–110.12	4.897	0.013*	0.197^ȶȶȶ^	5.294	0.009*	0.209^ȶȶȶ^
PM	Mid-test	107.23 (9.14)§	103.07–111.39	106.29 (6.64)	103.27–109.32						
	Post-test	111.08 (7.53)	107.65–114.51	107.10 (7.79)	103.55–110.65						
	Pre-test	101.10 (9.37)	96.83–105.37	101.10 (7.18)	97.82–104.37	2.401	0.104	0.107^ȶȶ^	5.061	0.011*	0.202^ȶȶȶ^
PL	Mid-test	101.14 (9.66)§	96.74–105.54	100.45 (8.72)	96.48–104.42						
	Post-test	104.94 (7.15)	101.68–108.20	101.93 (8.33)	98.13–105.72						
	Pre-test	94.65 (5.81)^§^	92.00–97.30	94.58 (5.07)	92.27–96.88	9.802	<0.001*	0.329^ȶȶȶ^	5.679	0.013*	0.221^ȶȶȶ^
Composite	Mid-test	94.57 (6.80)^§^	91.47–97.67	94.54 (5.69)	91.94–97.13						
	Post-test	97.88 (4.52)	95.82–99.94	94.71 (5.89)	92.03–97.39						
	Pre-test	31.80 (18.82)^§^	22.99–40.61	35.24 (15.55)	27.96–42.53	4.012	0.026*	0.174^ȶȶȶ^	10.176	<0.001*	0.349^ȶȶȶ^
SLST (sec)	Mid-test	31.88 (18.80)^§^	23.07–40.68	31.62 (17.31)	23.51–39.72						
	Post-test	42.87 (18.37)	34.27–51.47	34.77 (18.98)	25.89–43.65						
	Pre-test	158.95 (23.33)^§^	148.33–169.57	152.04 (22.54)	141.78–162.30	8.972	<0.001*	0.310^ȶȶȶ^	8.172	0.001*	0.290^ȶȶȶ^
SLHD (%)	Mid-test	154.56 (24.32)^§^	143.49–165.33	151.10 (24.94)	139.75–162.46						
	Post-test	167.33 (19.32)	158.53–176.12	151.57 (23.73)	140.77–162.37						
	Pre-test	35.87 (7.22)^†^	32.58–39.16	35.99 (8.16)^†§^	32.28–39.71	2.026	0.145	0.092^ȶȶ^	13.721	<0.001*	0.407^ȶȶȶ^
CMJ (cm)	Mid-test	33.64 (8.12)	29.94–37.34	33.87 (8.10)	30.18–37.56						
*	Post-test	34.41 (7.93)	30.80–38.02	32.60 (7.47)	29.20–36.00						

Note: *FR,* foam rolling group; *PR,* passive recovery group; *YBT*, normalized y-balance test; *ANT,* anterior; *PM,* posteromedial; *PL*, posterolateral; *SLST*, single-leg stance test; *CMJ*, countermovement jump; *SLHD*, normalized single‑leg hop for distance; †, significantly different from the mid-test (p < .05); §, significantly different from the post-test (p < .05); bold and * indicate a statistically significant difference (p < .05); ${\eta }_{p}^{2}$, effect size magnitudes; ȶ, small effect, ȶȶ, moderate effect, ȶȶȶ, large effect.

### Physical outcomes

Considering the physical performance outcomes, significant main effects of time and intervention × time interactions with large effect sizes were observed for the YBT composite score, YBT-PM direction, SLST, and SLHD (see Table 3). In the YBT-PL direction and CMJ, significant main effects of time with large effect sizes were observed, while no significant intervention × time interactions with moderate effect sizes were found. For the YBT-ANT direction, neither significant main effects of time nor intervention × time interactions with moderate effect sizes were observed.

In pairwise comparisons, significant time effects with predominantly moderate magnitudes were observed for: 1) YBT-PM, YBT composite score, SLST, and SLHD from pre-test to post-test and mid-test to post-tests (d^z^ = 0.69–1.04) in the FR group; 2) YBT-PL from mid-test to post-test (d^z^ = 0.76) in the FR group; 3) CMJ from pre-test to mid-test (d^z^ = 0.66) in the FR group while from pre-test to mid-test and from pre-test to post-test (d^z^ = 0.81–1.07) in the PR group (see Table 3 and Fig. 3).

### sEMG findings

Regarding sEMG excitations, significant main effects of time, with moderate-to-large magnitudes, were observed for YBT-ANT (RF, VM, BF), YBT-PM (RF, VM, BF, ST), YBT-PL (RF, VM, VL, BF), SLST (RF, VM, VL), and SLHD (RF, GM). No significant main effects of time were observed for CMJ (small-to-moderate magnitudes). Additionally, no significant intervention × time interactions were found for any measure (small-to-moderate magnitudes) (see S1).

In pairwise comparisons, significant time effects with predominantly moderate-to-large magnitudes were observed for: (1) YBT-ANT for RF and BF from pre-test to mid-test and post-test in both groups (d^z^ = 0.57–1.14), and for VM from pre-test to mid-test (d^z^ = 0.80), in the PR group; (2) YBT-PM for RF from pre-test to mid-test and post-test (d^z^ = 0.57–0.81) in both groups, for VM from pre-test to mid-test (d^z^ = 0.95) in the PR group, and for BF from pre-test to mid-test and mid-test to post-test (d^z^ = 0.64–1.02) in the PR group; (3) YBT-PL for RF and VM from pre-test to post-test (d^z^ = 0.68–0.93) in the FR group, for RF from pre-test to mid-test and pre-test to post-test (d^z^ = 0.58–0.63) in the PR group, and for VM and BF from pre-test to mid-test in the PR group(d^z^ = 0.77–1.43); (4) SLST for RF from mid-test to post-test in the PR group (d^z^ = 0.64), and for VM and VL from pre-test to mid-test (d^z^ = 0.59–0.69) in the PR group; (5) SLHD for RF from mid-test to post-test (d^z^ = 0.59) in the PR group; and (6) no significant time effects were observed for CMJ (see S1 File and Fig. 4).

## Discussion

In this study, we compared the acute effects of FR and PR following a single bout of CCT on YBT, SLST, SLHD, and CMJ performances, as well as sEMG activities. The main findings indicate that FR, compared with PR, significantly supported dynamic balance, static balance, and horizontal jump performance after a single bout of CCT. However, FR did not produce a significant advantage in vertical jump performance. Another important finding was that, although FR led to alterations (increases or decreases) in muscle excitation at certain time points, the overall effect was limited, and no consistent excitation trend was observed.

Regarding YBT performance, the FR intervention improved overall dynamic balance performance compared with PR. In terms of muscle excitation, no consistent direction of change was observed following FR. This suggests that FR did not produce a marked increase in the total muscle excitation level (based on normalized MVIC values) and that improvements in dynamic performance may not be directly related to increased excitation (MacDonald et al. 2013; Borisavljević et al. 2025). This may suggest that FR could potentially influence dynamic balance performance by slightly enhancing muscle control, possibly through changes in the viscoelastic properties (e.g., thixotropic effects) of the muscle tendon complex, or by modestly affecting proprioceptive feedback (Konrad et al. 2022a; Seever et al. 2022). Indeed, Borisavljević et al. (2025) reported that a short FR duration (30 s) led to a transient rise in sEMG activity, while longer durations (15–60 s) tended to reduce excitation without affecting maximal efficiency. This implies that the muscle could generate similar force with lower excitation, reflecting greater neuromuscular efficiency. In line with these findings, the present observation that FR improved performance at similar sEMG levels may suggest a potential enhancement in neuromuscular efficiency, although this interpretation should be considered speculative (Bradbury-Squires et al. 2015; Macgregor et al. 2018). However, it should be noted that sEMG amplitude alone may not reliably represent physical performance.

Considering SLST performance, FR produced a significant improvement compared with PR. sEMG results revealed no significant differences in lower-limb muscle excitation except for the quadriceps (e.g., RF). However, Mandalidis and Karagiannakis (2020) reported that gastrocnemius muscles, rather than the quadriceps, are the main controllers of posture during SLST, consistent with our results (see Fig. 4). Moreover, several studies have shown a positive association between improved proprioceptive feedback and enhanced static balance performance. For example, David et al. (2019) found that FR applied to the lower limbs enhanced knee and hip joint proprioception, while Naderi et al. (2020) reported that FR accelerated the recovery of proprioception after intense exercise. Therefore, the observed improvement in static balance performance in our study may be attributed to enhanced proprioceptive feedback induced by FR. Additionally, reductions in muscle stiffness and improved flexibility may have helped optimize muscle tone and facilitate motor control, thus supporting static balance (Nakamura et al. 2021; Konrad et al. 2021; Rodoplu et al. 2025). The absence of significant differences in muscle excitation between recovery conditions aligns with previous research showing that FR does not directly increase excitation but may help maintain existing levels by reducing intramuscular pain and inflammation, thereby limiting neural inhibition (MacDonald et al. 2013, 2014). This mechanism may relate to reduced neural suppression following exercise-induced muscle damage. Thus, even though FR did not significantly alter excitation, improvements in proprioceptive feedback and muscle tone likely contributed to enhanced static balance performance. Finally, hip abductor muscles (particularly the gluteus medius) also play a role in static balance; therefore, future studies should take these muscles into account (Porto et al. 2019).

Regarding SLHD, FR acutely improved performance but did not cause significant changes in muscle excitation. In contrast to our results, Junker and Stöggl (2019) reported that FR did not improve horizontal jump or triple-hop performance. However, the results of both our study and theirs found no significant differences in sEMG excitation, suggesting consistent findings in this regard. The discrepancy in performance outcomes may be due to differences in study design, such as Junker and Stöggl focusing only on the hamstrings and using a long-term (8-week) intervention. Meanwhile, Sullivan et al. (2013) found that FR improved ROM but had no significant effect on muscle excitation. These findings suggest that FR may enhance movement efficiency by improving the viscoelastic properties of the muscle–tendon unit (Behara and Jacobson 2017) and that increased flexibility may particularly support horizontal jump performance (Wu et al. 2020). Overall, the acute effects of FR may more likely be related to improvements in muscle function and motor control mechanisms than from direct changes in neuromuscular excitation, consistent with previous research (Junker and Stöggl 2019; Wiewelhove et al. 2019).

Our findings considering CMJ indicate that FR did not have a significant impact on vertical jump performance or muscle excitation. This aligns with previous studies reporting inconsistent effects of FR on power and explosive performance measures such as vertical jumps (Laffaye et al. 2019; Wiewelhove et al. 2019). Similarly, our previous study (Rodoplu et al. 2025) found that FR following strength exercise did not alter CMJ performance in the short term. These findings suggest that exercise-induced fatigue may limit muscle force production through both central (e.g., muscle excitation, central drive) and peripheral (e.g., excitation–contraction coupling, metabolite accumulation, myofibrillar damage) mechanisms, which short FR applications may not immediately reverse. Furthermore, the absence of significant changes in muscle excitation during CMJ (particularly in the propulsion phase) is consistent with prior literature (Bobbert et al. 1996; Rodríguez-Rosell, 2021), suggesting that FR may primarily produce sensory or local tissue effects in the short term, with limited potential to enhance motor unit behaviour or performance in high-velocity, stretch–shortening cycle (SSC) tasks. Consequently, FR applied alone after high-intensity exercise may not acutely enhance CMJ performance; future research could explore its combination with other recovery strategies (Konrad et al. 2022a, b; Rodoplu et al. 2025).

Interestingly, while FR improved SLHD performance, no similar effect was observed for CMJ, and no significant differences were found in muscle excitation across both tests. This may stem from the distinct biomechanical characteristics of the two tests (Bobbert et al. 1996; Pilanthananond et al. 2023). CMJ is a double-leg, vertically oriented explosive movement, whereas SLHD involves a forward-directed, single-leg push, thus reflecting different movement patterns and muscle demands. In this context, anterior chain muscles such as RF, VM, and VL may play a greater role in CMJ than in SLHD, consistent with our data (see Fig. 4). Moreover, literature suggests that horizontal jumps like SLHD involve longer and slower stretch–shortening cycles, allowing greater muscle excitation by the BF (Nygaard Falch et al. 2020). These biomechanical distinctions likely explain why FR improved SLHD but not CMJ performance. In summary, the effects of FR on performance likely occur indirectly through enhanced proprioceptive sensitivity, dynamic balance control, or altered mechanical properties (e.g., stiffness/flexibility) of the muscle–tendon unit, rather than through direct increases in muscle excitation.

The practical relevance of the present findings should be interpreted in the context of performance preservation and recovery support rather than absolute performance enhancement. In particular, in applied sport environments characterized by multiple training sessions within the same day, intensive training camps, tournament-style competition periods, or limited recovery windows, attenuating acute fatigue-related performance decrements may be functionally meaningful. In these contexts, the preservation of balance and single-leg hop performance may be relevant not only for maintaining performance continuity but also for sustaining neuromuscular control, preserving postural stability, and managing potential injury risk. Accordingly, the effects attributed to foam rolling in the present study should be considered not as a performance-enhancing intervention per se, but as a recovery-supporting approach that may contribute to the acute preservation of functional performance following high-demand training sessions.

This study has several limitations. First, only male participants were included, limiting the generalizability of the findings. Future studies should include female participants, especially when analyzing muscle excitation outcomes. Additionally, not including more lower-limb muscles (e.g., soleus, tibialis anterior, gluteal muscles) in sEMG recordings during balance and jump tests restricted a more comprehensive assessment of excitation patterns. Future research should address this by including additional muscle groups. Moreover, the absence of force-platform (e.g., CoP) data limited the detailed analysis of postural control strategies, and including such measures in future studies would be valuable. In addition, muscle–tendon stiffness was not assessed, and incorporating this parameter could provide further insight into potential mechanical adaptations. Future research should include these variables and examine different FR durations, intensities, and comparisons with other recovery modalities to better understand the effectiveness of FR.

## Conclusion

Following a single bout of CCT, FR acutely improved dynamic balance (e.g., YBT), static balance (e.g., SLST), and horizontal jump (e.g., SLHD) performances compared with PR, while vertical jump (e.g., CMJ) performance was not significantly affected. The sEMG data did not show a consistent increase in muscle excitation across the assessed muscles, although some trends were observed, suggesting that the performance improvements are likely related to other mechanisms such as enhanced proprioception, changes in the viscoelastic properties of the muscle–tendon unit, and potential neuromuscular efficiency. From a practical perspective, FR may be useful for attenuating fatigue-induced decrements and providing measurable, task-specific improvements in dynamic and static balance as well as horizontal jump performance, whereas additional strategies may be needed to improve vertical jump under the conditions and protocol of the present study. Future studies should include a more comprehensive sEMG setup, center of pressure (CoP) analyses, and different FR protocols could provide further insight into the underlying mechanisms and optimize performance outcomes.

## Supplementary Information

Below is the link to the electronic supplementary material.

Supplementary file1 (DOCX 60 KB)

## Data Availability

The data analyzed during the current study are accessible from the corresponding author on reasonable request.

## Abbreviations

ANOVA: Analysis of variance
CCT: Complex contrast training
CMJ: Countermovement jump
FR: Foam rolling
MVIC: Maximum voluntary isometric contraction
PR: Passive recovery
sEMG: Surface electromyography
SLHD: Single-leg hop for distance
SLST: Single-leg stance test
YBT: Y-balance test
