# Supplementary material for: Acute effects of foam rolling vs. passive rest following a single bout of complex contrast training
Source: Eur J Appl Physiol. 2026 Mar 24;126(7):3941–54. doi: 10.1007/s00421-026-06188-8 (PMC13380571; doi:10.1007/s00421-026-06188-8)
Supplement: Supplementary file 1 — Supplementary file1 (DOCX 60 KB) [file 421_2026_6188_MOESM1_ESM.docx]

| **Supplementary File 1.**  Participants’ EMG Activities Normalized to MVC: Means, Confidence Intervals, and ANOVA Analysis | | | | | | | | | | | | | | | |
| --- | --- | --- | --- | --- | --- | --- | --- | --- | --- | --- | --- | --- | --- | --- | --- |
| **Variable** | **Time** | **FR** | |  | **PR** | |  | **Intervention X Time** | | |  | **Time** | | | |
|  |  | **Mean (SD)** | **[95% CI]** |  | **Mean (SD)** | **[95% CI]** |  | **F _(2, 40)_** | ***p*** | $\boldsymbol{\eta}_{\boldsymbol{p}}^{\boldsymbol{2}}$ |  | **F _(2, 40)_** | ***p*** | $\boldsymbol{\eta}_{\boldsymbol{p}}^{\boldsymbol{2}}$ |  |
| **YBT-ANT** | Pre-test | 17.31 (9.79) **†§** | 12.86 – 21.77 |  | 16.66 (8.01) **†§** | 13.01 – 20.31 |  | 1.212 | .308 | .057 ^ȶ^ |  | 17.182 | **<.001*** | .462 ^ȶȶȶ^ | |
| *RF* | Mid-test | 20.30 (10.52) | 15.51 – 25.09 |  | 21.70 (9.50) | 17.37 – 26.03 |  |  |  |  |  |  |  |  |  |
|  | Post-test | 20.83 (9.91) | 16.32 – 25.35 |  | 19.92 (10.39) | 15.19 – 24.65 |  |  |  |  |  |  |  |  |  |
|  | Pre-test | 32.69 (10.89) | 27.73 – 37.65 |  | 33.73 (11.07) **†** | 28.69 – 38.78 |  | 2.393 | .104 | .107 ^ȶȶ^ |  | 7.203 | **.002*** | .265 ^ȶȶȶ^ |  |
| *VM* | Mid-test | 35.37 (10.29) | 30.69 – 40.06 |  | 41.48 (16.32) | 34.05 – 48.91 |  |  |  |  |  |  |  |  |  |
|  | Post-test | 36.46 (11.42) | 31.26 – 41.66 |  | 37.90 (14.05) | 31.50 – 44.30 |  |  |  |  |  |  |  |  |  |
|  | Pre-test | 35.80 (9.01) | 31.69 – 39.90 |  | 35.73 (10.19) | 31.09 – 40.37 |  | .445 | .630 | .022 ^ȶ^ |  | 3.119 | .070 | .135 ^ȶȶ^ |  |
| *VL* | Mid-test | 37.37 (9.05) | 33.25 – 41.49 |  | 37.70 (7.80) | 34.15 – 41.26 |  |  |  |  |  |  |  |  |  |
|  | Post-test | 40.49 (14.87) | 33.72 – 47.26 |  | 38.41 (12.46) | 32.74 – 44.09 |  |  |  |  |  |  |  |  |  |
|  | Pre-test | 7.33 (4.16) **†§** | 5.44 – 9.23 |  | 8.97 (6.73) **†§** | 5.91 – 12.04 |  | 1.394 | .260 | .065 ^ȶȶ^ |  | 10.535 | **<.001*** | .345 ^ȶȶȶ^ |  |
| *BF* | Mid-test | 8.81 (4.57) | 6.73 – 10.90 |  | 10.51 (6.33) | 7.63 – 13.40 |  |  |  |  |  |  |  |  |  |
|  | Post-test | 9.56 (5.10) | 7.24 – 11.89 |  | 10.13 (6.28) | 7.27 – 12.99 |  |  |  |  |  |  |  |  |  |
|  | Pre-test | 13.41 (8.82) | 9.39 – 17.43 |  | 13.76 (9.17) | 9.59 – 17.94 |  | 2.927 | .065 | .128 ^ȶȶ^ |  | 1.086 | .347 | .051 ^ȶ^ |  |
| *ST* | Mid-test | 15.39 (9.59) | 11.02 – 19.76 |  | 13.34 (8.59) | 9.43 – 17.26 |  |  |  |  |  |  |  |  |  |
|  | Post-test | 13.72 (9.17) | 9.64 – 17.80 |  | 13.39 (9.59) | 9.02 – 17.76 |  |  |  |  |  |  |  |  |  |
|  | Pre-test | 11.14 (4.40) | 9.13 – 13.14 |  | 11.68 (4.04) | 9.84 – 13.52 |  | 1.030 | .366 | .049 ^ȶ^ |  | .021 | .979 | .001 ^ȶ^ |  |
| *GM* | Mid-test | 11.05 (4.63) | 8.94 – 13.16 |  | 11.82 (4.39) | 9.82 – 13.82 |  |  |  |  |  |  |  |  |  |
|  | Post-test | 11.53 (3.87) | 9.77 – 13.30 |  | 11.14 (3.94) | 9.34 – 12.93 |  |  |  |  |  |  |  |  |  |
| **YBT-PM** | Pre-test | 28.07 (12.33) **†§** | 22.45 – 33.68 |  | 27.50 (12.47) **†§** | 21.82 – 33.18 |  |  |  |  |  |  |  |  |  |
| *RF* | Mid-test | 32.48 (14.56) | 25.85 – 39.11 |  | 32.00 (14.18) | 25.55 – 38.46 |  | .006 | .994 | .000 ^ȶ^ |  | 8.362 | **<.001*** | .295 ^ȶȶȶ^ |  |
|  | Post-test | 32.72 (11.60) | 26.11 – 37.88 |  | 32.00 (12.92) | 26.11 – 37.88 |  |  |  |  |  |  |  |  |  |
|  | Pre-test | 34.28 (9.03) | 30.17 – 38.39 |  | 37.40 (12.51) **†** | 31.71 – 43.10 |  |  |  |  |  |  |  |  |  |
| *VM* | Mid-test | 41.24 (15.05) | 34.38 – 48.09 |  | 45.57 (15.06) | 38.71 – 52.43 |  | .339 | .715 | .017 ^ȶ^ |  | 11.265 | **<.001*** | .360 ^ȶȶȶ^ |  |
|  | Post-test | 39.49 (12.71) | 33.70 – 45.28 |  | 41.07 (12.50) | 35.37 – 46.76 |  |  |  |  |  |  |  |  |  |
|  | Pre-test | 38.23 (9.73) | 33.80 – 42.66 |  | 38.59 (9.94) | 34.06 – 43.11 |  |  |  |  |  |  |  |  |  |
| *VL* | Mid-test | 40.24 (10.34) | 35.54 – 44.95 |  | 41.66 (7.98) | 38.02 – 45.29 |  | 1.670 | .205 | .077 ^ȶȶ^ |  | 2.265 | .134 | .102 ^ȶȶ^ |  |
|  | Post-test | 43.27 (13.41) | 37.16 – 49.38 |  | 40.10 (11.07) | 35.06 – 45.14 |  |  |  |  |  |  |  |  |  |
|  | Pre-test | 5.78 (2.19) | 4.78 – 6.78 |  | 6.21 (2.79) **†** | 4.94 – 7.49 |  |  |  |  |  |  |  |  |  |
| *BF* | Mid-test | 6.80 (3.29) | 5.30 – 8.30 |  | 7.58 (3.52) **§** | 5.97 – 9.19 |  | .493 | .615 | .024 ^ȶ^ |  | 7.224 | **.006*** | .265 ^ȶȶȶ^ |  |
|  | Post-test | 6.71 (3.63) | 5.06 – 8.37 |  | 6.98 (3.60) | 5.34 – 8.62 |  |  |  |  |  |  |  |  |  |
|  | Pre-test | 7.36 (3.64) | 5.70 – 9.01 |  | 6.41 (2.62) | 5.21 – 7.61 |  |  |  |  |  |  |  |  |  |
| *ST* | Mid-test | 8.39 (4.40) **§** | 6.39 – 10.39 |  | 6.78 (2.57) | 5.61 – 7.95 |  | 1.011 | .373 | .048 ^ȶ^ |  | 4.693 | **.015*** | .190 ^ȶȶȶ^ |  |
|  | Post-test | 7.07 (3.37) | 5.53 – 8.61 |  | 6.25 (2.38) | 5.17 – 7.34 |  |  |  |  |  |  |  |  |  |
|  | Pre-test | 10.34 (4.30) | 8.38 – 12.30 |  | 10.54 (4.13) | 8.66 – 12.43 |  |  |  |  |  |  |  |  |  |
| *GM* | Mid-test | 9.99 (4.64) | 7.88 – 12.11 |  | 10.66 (4.01) | 8.83 – 12.49 |  | .238 | .789 | .012 ^ȶ^ |  | .474 | .626 | .023 ^ȶ^ |  |
|  | Post-test | 10.30 (4.11) | 8.43 – 12.18 |  | 11.06 (3.99) | 9.24 – 12.88 |  |  |  |  |  |  |  |  |  |
| **YBT-PL** | Pre-test | 21.62 (8.47) **§** | 17.77 – 25.48 |  | 21.68 (9.57) **†§** | 17.32 – 26.03 |  |  |  |  |  |  |  |  |  |
| *RF* | Mid-test | 25.79 (10.42) | 21.04 – 30.53 |  | 25.43 (10.61) | 20.60 – 30.27 |  | .526 | .595 | .026 ^ȶ^ |  | 12.140 | **<.001*** | .378 ^ȶȶȶ^ |  |
|  | Post-test | 28.62 (10.10) | 24.02 – 33.22 |  | 26.54 (11.76) | 21.19 – 31.90 |  |  |  |  |  |  |  |  |  |
|  | Pre-test | 33.50 (11.19) **§** | 28.41 – 38.60 |  | 40.54 (17.19) **†** | 32.71 – 48.73 |  |  |  |  |  |  |  |  |  |
| *VM* | Mid-test | 39.67 (17.11) | 31.88 – 47.46 |  | 48.77 (19.25) | 40.00 – 57.53 |  | 1.402 | .258 | .065 ^ȶȶ^ |  | 13.467 | **<.001*** | .402 ^ȶȶȶ^ |  |
|  | Post-test | 41.22 (16.34) | 33.78 – 48.66 |  | 45.22 (17.31) | 37.34 – 53.10 |  |  |  |  |  |  |  |  |  |
|  | Pre-test | 35.89 (7.24) **§** | 32.59 – 39.19 |  | 36.43 (10.24) | 31.77 – 41.10 |  |  |  |  |  |  |  |  |  |
| *VL* | Mid-test | 38.34 (10.64) | 33.50 – 43.19 |  | 42.06 (13.26) | 36.03 – 48.10 |  | 2.934 | .065 | .128 ^ȶ^ |  | 4.460 | **.018*** | .182 ^ȶȶȶ^ |  |
|  | Post-test | 44.70 (17.35) | 36.81 – 52.61 |  | 40.00 (13.72) | 33.76 – 46.25 |  |  |  |  |  |  |  |  |  |
|  | Pre-test | 9.86 (6.97) | 6.68 – 13.03 |  | 10.00 (5.66) **†** | 7.43 – 12.58 |  |  |  |  |  |  |  |  |  |
| *BF* | Mid-test | 11.15 (8.28) | 7.38 – 14.92 |  | 12.60 (7.99) | 8.96 – 16.24 |  | 1.436 | .250 | .067 ^ȶȶ^ |  | 5.169 | .**010*** | .205 ^ȶȶȶ^ |  |
|  | Post-test | 10.04 (6.68) | 7.00 – 13.08 |  | 10.91 (5.70) | 8.32 – 13.51 |  |  |  |  |  |  |  |  |  |
|  | Pre-test | 5.93 (2.69) | 4.71 – 7.16 |  | 5.73 (2.28) | 4.69 – 6.77 |  |  |  |  |  |  |  |  |  |
| *ST* | Mid-test | 6.79 (3.21) | 5.33 – 8.25 |  | 6.29 (2.19) | 5.29 – 7.28 |  | .869 | .404 | .042 ^ȶ^ |  | 3.016 | .076 | .131 ^ȶȶ^ |  |
|  | Post-test | 6.68 (3.01) | 5.31 – 8.05 |  | 5.73 (2.29) | 4.69 – 6.78 |  |  |  |  |  |  |  |  |  |
|  | Pre-test | 13.58 (7.38) | 10.22 – 16.93 |  | 15.61 (6.47) | 12.66 – 18.56 |  |  |  |  |  |  |  |  |  |
| *GM* | Mid-test | 12.99 (6.33) | 10.11 – 15.87 |  | 14.56 (5.69) | 11.97 – 17.15 |  | 1.545 | .226 | .072 ^ȶ^ |  | 1.289 | .287 | .061 ^ȶȶ^ |  |
|  | Post-test | 13.40 (4.47) | 11.37 – 15.44 |  | 13.10 (5.02) | 10.81 – 15.38 |  |  |  |  |  |  |  |  |  |
| **SLST** | Pre-test | 3.90 (2.94) | 2.56 – 5.24 |  | 5.19 (5.35) | 2.75 – 7.62 |  |  |  |  |  |  |  |  |  |
| *RF* | Mid-test | 3.04 (2.47) | 1.91 – 4.16 |  | 3.14 (2.31) **§** | 2.09 – 4.19 |  | 1.203 | .310 | .057 ^ȶ^ |  | 5.233 | **.021*** | .207 ^ȶȶȶ^ |  |
|  | Post-test | 3.21 (2.45) | 2.10 – 4.33 |  | 4.02 (3.02) | 2.64 – 5.40 |  |  |  |  |  |  |  |  |  |
|  | Pre-test | 6.85 (4.04) | 5.00 – 8.69 |  | 7.47 (4.62) **†** | 5.37 – 9.58 |  |  |  |  |  |  |  |  |  |
| *VM* | Mid-test | 5.56 (4.06) | 3.71 – 7.41 |  | 5.41 (3.63) | 3.76 – 7.06 |  | 1.075 | .351 | .051 ^ȶ^ |  | 4.837 | **.013*** | .195 ^ȶȶȶ^ |  |
|  | Post-test | 5.07 (3.41) | 3.52 – 6.62 |  | 6.20 (3.67) | 4.53 – 7.88 |  |  |  |  |  |  |  |  |  |
|  | Pre-test | 5.14 (3.40) | 3.59 – 6.69 |  | 5.50 (3.52) **†** | 3.90 – 7.10 |  |  |  |  |  |  |  |  |  |
| *VL* | Mid-test | 4.11 (3.02) | 2.73 – 5.48 |  | 4.00 (2.32) | 2.94 – 5.05 |  | .198 | .821 | .010 ^ȶ^ |  | 4.219 | **.022*** | .174 ^ȶȶȶ^ |  |
|  | Post-test | 4.29 (3.34) | 2.77 – 5.81 |  | 4.50 (2.77) | 3.24 – 5.76 |  |  |  |  |  |  |  |  |  |
|  | Pre-test | 3.23 (2.10) | 2.28 – 4.19 |  | 4.12 (4.44) | 2.10 – 6.14 |  |  |  |  |  |  |  |  |  |
| *BF* | Mid-test | 4.78 (3.50) | 3.18 – 6.37 |  | 5.67 (6.26) | 2.83 – 8.52 |  | .006 | .978 | .000 ^ȶ^ |  | 2.580 | .088 | .114 ^ȶȶ^ |  |
|  | Post-test | 4.02 (3.63) | 2.37 – 5.67 |  | 4.86 (4.76) | 2.69 – 7.03 |  |  |  |  |  |  |  |  |  |
|  | Pre-test | 4.42 (3.07) | 3.02 – 5.81 |  | 5.14 (3.95) | 3.34 – 6.94 |  |  |  |  |  |  |  |  |  |
| *ST* | Mid-test | 5.85 (4.10) | 3.98 – 7.71 |  | 5.72 (3.94) | 3.93 – 7.52 |  | 1.101 | .342 | .052 ^ȶ^ |  | 2.557 | .089 | .119 ^ȶȶ^ |  |
|  | Post-test | 4.80 (3.71) | 3.11 – 6.48 |  | 5.34 (3.90) | 3.57 – 7.11 |  |  |  |  |  |  |  |  |  |
|  | Pre-test | 20.24 (7.54) | 16.81 – 23.67 |  | 23.27 (8.82) | 19.26 – 27.29 |  |  |  |  |  |  |  |  |  |
| *GM* | Mid-test | 20.06 (6.94) | 16.90 – 23.22 |  | 21.49 (6.35) | 18.60 – 24.37 |  | 1.083 | .348 | .051 ^ȶ^ |  | 2.628 | .085 | .116 ^ȶȶ^ |  |
|  | Post-test | 21.73 (6.82) | 18.63 – 24.84 |  | 22.92 (8.27) | 19.16 – 26.69 |  |  |  |  |  |  |  |  |  |
| **SLHD** | Pre-test | 51.16 (18.22) | 42.87 – 59.45 |  | 46.70 (18.16) | 38.43 – 54.96 |  |  |  |  |  |  |  |  |  |
| *RF* | Mid-test | 54.34 (15.49) | 47.29 – 61.39 |  | 51.10 (22.10) **§** | 41.05 – 61.16 |  | .516 | .601 | .025 ^ȶ^ |  | 3.457 | **.041*** | .147 ^ȶȶȶ^ |  |
|  | Post-test | 51.30 (15.50) | 44.25 – 58.36 |  | 44.58 (18.61) | 36.11 – 53.05 |  |  |  |  |  |  |  |  |  |
|  | Pre-test | 57.44 (20.80) | 47.98 – 66.91 |  | 59.63 (26.91) | 47.39 – 71.88 |  |  |  |  |  |  |  |  |  |
| *VM* | Mid-test | 55.32 (18.56) | 46.87 – 63.77 |  | 56.98 (21.32) | 47.28 – 66.69 |  | 1.609 | .213 | .074 ^ȶȶ^ |  | .671 | .517 | .032 ^ȶ^ |  |
|  | Post-test | 58.59 (22.89) | 48.17 – 69.01 |  | 53.76 (17.12) | 45.97 – 61.55 |  |  |  |  |  |  |  |  |  |
|  | Pre-test | 61.72 (21.41) | 51.97 – 71.47 |  | 60.17 (21.87) | 50.22 – 70.13 |  |  |  |  |  |  |  |  |  |
| *VL* | Mid-test | 57.88 (21.03) | 48.31 – 67.45 |  | 59.36 (18.56) | 50.91 – 67.80 |  | 1.477 | .240 | .069 ^ȶȶ^ |  | .520 | .598 | .025 ^ȶ^ |  |
|  | Post-test | 64.36 (25.31) | 52.84 – 75.88 |  | 57.80 (20.40) | 48.51 – 67.08 |  |  |  |  |  |  |  |  |  |
|  | Pre-test | 29.40 (14.04) | 23.01 – 35.79 |  | 33.33 (16.18) | 25.97 – 40.70 |  |  |  |  |  |  |  |  |  |
| *BF* | Mid-test | 30.74 (18.18) | 22.46 – 39.01 |  | 32.43 (17.58) | 24.43 – 40.44 |  | .805 | .454 | .039 ^ȶ^ |  | .415 | .663 | .020 ^ȶ^ |  |
|  | Post-test | 30.37 (16.24) | 22.98 – 37.76 |  | 29.62 (14.73) | 22.92 – 36.33 |  |  |  |  |  |  |  |  |  |
|  | Pre-test | 16.26 (9.22) | 12.07 – 20.46 |  | 16.91 (12.91) | 11.03 – 22.79 |  |  |  |  |  |  |  |  |  |
| *ST* | Mid-test | 16.78 (9.62) | 12.40 – 21.16 |  | 16.26 (12.58) | 10.53 – 21.99 |  | .622 | .542 | .030 ^ȶ^ |  | .037 | .964 | .002 ^ȶ^ |  |
|  | Post-test | 17.95 (11.59) | 12.67 – 23.22 |  | 15.62 (13.39) | 9.52 – 21.72 |  |  |  |  |  |  |  |  |  |
|  | Pre-test | 78.13 (17.40) | 70.21 – 86.05 |  | 83.04 (16.42) | 75.57 – 90.52 |  |  |  |  |  |  |  |  |  |
| *GM* | Mid-test | 84.86 (14.63) | 78.20 – 91.52 |  | 86.70 (13.67) | 80.48 – 92.92 |  | 1.722 | .192 | .079 ^ȶȶ^ |  | 3.372 | **.044*** | .144 ^ȶȶȶ^ |  |
|  | Post-test | 87.59 (15.76) | 80.42 – 94.76 |  | 84.46 (13.13) | 78.48 – 90.44 |  |  |  |  |  |  |  |  |  |
| **CMJ** | Pre-test | 77.28 (19.00) | 68.63 – 85.93 |  | 67.05 (19.52) | 58.16 – 75.93 |  |  |  |  |  |  |  |  |  |
| *RF* | Mid-test | 73.22 (17.71) | 65.16 – 81.28 |  | 69.27 (26.85) | 57.05 – 81.49 |  | 1.126 | .334 | .053 ^ȶ^ |  | .092 | .913 | .005 ^ȶ^ |  |
|  | Post-test | 73.57 (18.67) | 65.07 – 82.07 |  | 69.50 (26.28) | 57.54 – 81.47 |  |  |  |  |  |  |  |  |  |
|  | Pre-test | 75.20 (18.60) | 66.74 – 83.67 |  | 72.78 (21.21) | 63.12 – 82.43 |  |  |  |  |  |  |  |  |  |
| *VM* | Mid-test | 70.02 (20.77) | 60.57 – 79.48 |  | 77.92 (22.60) | 67.63 – 88.20 |  | 3.016 | .076 | .131 ^ȶȶ^ |  | .002 | .998 | .000 ^ȶ^ |  |
|  | Post-test | 74.52 (21.66) | 64.66 – 84.38 |  | 73.67 (18.65) | 65.18 – 82.16 |  |  |  |  |  |  |  |  |  |
|  | Pre-test | 76.51 (20.60) | 67.13 – 85.89 |  | 77.37 (21.93) | 67.39 – 87.36 |  |  |  |  |  |  |  |  |  |
| *VL* | Mid-test | 75.06 (22.07) | 65.02 – 85.11 |  | 73.91 (18.34) | 65.56 – 82.26 |  | .363 | .675 | .018 ^ȶ^ |  | .714 | .457 | .034 ^ȶ^ |  |
|  | Post-test | 75.76 (25.75) | 64.04 – 87.48 |  | 72.00 (19.40) | 63.17 – 80.83 |  |  |  |  |  |  |  |  |  |
|  | Pre-test | 26.30 (12.09) | 20.79 – 31.80 |  | 23.41 (11.12) | 18.35 – 28.47 |  |  |  |  |  |  |  |  |  |
| *BF* | Mid-test | 29.10 (19.20) | 20.36 – 37.84 |  | 26.54 (14.42) | 19.97 – 33.10 |  | .478 | .624 | .023 ^ȶ^ |  | 1.705 | .195 | .079 ^ȶȶ^ |  |
|  | Post-test | 27.32 (17.72) | 19.25 – 35.38 |  | 26.59 (17.42) | 18.66 – 34.52 |  |  |  |  |  |  |  |  |  |
|  | Pre-test | 15.66 (10.45) | 10.91 – 20.42 |  | 12.75 (7.03) | 9.55 – 15.95 |  |  |  |  |  |  |  |  |  |
| *ST* | Mid-test | 16.99 (12.00) | 11.52 – 22.45 |  | 13.89 (8.45) | 10.05 – 17.74 |  | .424 | .658 | .021 ^ȶ^ |  | .814 | .450 | .039 ^ȶ^ |  |
|  | Post-test | 17.93 (15.35) | 10.94 – 24.92 |  | 13.22 (8.51) | 9.35 – 17.09 |  |  |  |  |  |  |  |  |  |
|  | Pre-test | 76.92 (18.23) | 68.62 – 85.22 |  | 83.41 (17.36) | 75.51 – 91.32 |  |  |  |  |  |  |  |  |  |
| *GM* | Mid-test | 82.10 (16.15) | 74.74 – 89.45 |  | 86.44 (19.22) | 77.69 – 95.19 |  | 1.212 | .308 | .057 ^ȶ^ |  | 2.526 | .093 | .112 ^ȶȶ^ |  |
|  | Post-test | 83.82 (16.77) | 76.19 – 91.45 |  | 83.29 (16.32) | 75.86 – 90.72 |  |  |  |  |  |  |  |  |  |
| **Note:** FR: foam rolling group; PR: passive recovery group; YBT: normalized y-balance test; ANT: anterior; PM: posteromedial; PL: posterolateral; SLST: single-leg stance test; CMJ: countermovement jump; SLHD: normalized single‑leg hop for distance; RF: rectus femoris; VM: vastus medialis; VL: vastus lateralis; BF: biceps femoris; ST: semitendinosus; GM: gastrocnemius medialis; **†**: significantly different from the mid-test (p < .05); **§:** significantly different from the post-test (p < .05); ***:** statistically significant difference (p < .05);  $\eta_{p}^{2}$*:* partial eta square (effect size magnitudes: ȶ: small effect, ȶȶ: moderate effect, ȶȶȶ: large effect). | | | | | | | | | | | | | | | |
|  | | | | | | | | | | | | | | | |
